# Fibrosis and expression of extracellular matrix proteins in human interventricular septum in aortic valve stenosis and regurgitation

**DOI:** 10.1007/s00418-024-02268-y

**Published:** 2024-02-12

**Authors:** David Sedmera, Alena Kvasilova, Adam Eckhardt, Petr Kacer, Martin Penicka, Matej Kocka, Dana Schindler, Ron Kaban, Radka Kockova

**Affiliations:** 1 Institute of Anatomy, First Faculty of Medicine, Charles University, U Nemocnice 3, 128 00 Prague, Czech Republic; 2 Institute of Physiology, The Czech Academy of Sciences, Videnska 1024, 142 00 Prague, Czech Republic; 3 Department of Cardiac Surgery, Third Faculty of Medicine, Charles University, Prague, Czech Republic; 4 University Hospital Kralovske Vinohrady, Prague, Czech Republic; 5 Cardiovascular Center Aalst, OLV Clinic, 9300 Aalst, Belgium; 6 Na Homolce Hospital, Roentgenova 37/2, 150 30 Prague, Czech Republic

**Keywords:** Valvular heart disease, Pressure overload, Fibrosis, Collagen, Fibronectin

## Abstract

Valvular heart disease leads to ventricular pressure and/or volume overload. Pressure overload leads to fibrosis, which might regress with its resolution, but the limits and details of this reverse remodeling are not known. To gain more insight into the extent and nature of cardiac fibrosis in valve disease, we analyzed needle biopsies taken from the interventricular septum of patients undergoing surgery for valve replacement focusing on the expression and distribution of major extracellular matrix protein involved in this process. Proteomic analysis performed using mass spectrometry revealed an excellent correlation between the expression of collagen type I and III, but there was little correlation with the immunohistochemical staining performed on sister sections, which included antibodies against collagen I, III, fibronectin, sarcomeric actin, and histochemistry for wheat germ agglutinin. Surprisingly, the immunofluorescence intensity did not correlate significantly with the gold standard for fibrosis quantification, which was performed using Picrosirius Red (PSR) staining, unless multiplexed on the same tissue section. There was also little correlation between the immunohistochemical markers and pressure gradient severity. It appears that at least in humans, the immunohistochemical pattern of fibrosis is not clearly correlated with standard Picrosirius Red staining on sister sections or quantitative proteomic data, possibly due to tissue heterogeneity at microscale, comorbidities, or other patient-specific factors. For precise correlation of different types of staining, multiplexing on the same section is the best approach.

**Supplementary Information:**

The online version contains supplementary material available at 10.1007/s00418-024-02268-y.

## Introduction

Cardiac fibrosis is commonly defined as excessive deposition of the extracellular matrix (ECM), mostly collagen, in the myocardial interstitial space (Frangogiannis 2021). While there are potentially numerous sources of this overproduction, the majority is produced by fibroblasts, or their sedentary counterparts, fibrocytes. This type of fibrosis should be distinguished from replacement fibrosis, resulting from myocyte death (typically after myocardial infarction) and scar formation (Schimmel et al. 2022). While a certain amount of collagen (generally up to 5%) is physiological (Rain et al. 2016), increased percentage results in increased wall stiffness and myocardial dysfunction. There are other types of fibrosis, usually associated with disease processes, such as subepicardial fibrosis in myocarditis, subendocardial fibrosis associated with endocarditis, or prenatal abnormal hemodynamics (Pesevski et al. 2018).

One reason for a diffusely increased amount of ECM in the myocardium is pressure overload (Berk et al. 2007). Higher amount of ECM leads to increased myocardial strain and myocyte hypertrophy (Green et al. 2016; Morishita et al. 2021); when diffusion limits and physiological plasticity are exceeded, the cardiomyocytes die and are replaced by extra fibrous tissue. Pressure overload in humans occurs in uncontrolled hypertension or stenotic valve disease and can be relatively easily modeled in experimental animals, such as transverse aortic constriction (Toischer et al. 2010) or renal artery clip (Loud et al. 1978), at different ages starting in the early postnatal period (Sedmera et al. 2003). Interestingly, while increased pressure load is a powerful fibrotic stimulus, volume overload, leading to similar levels of ventricular hypertrophy, is not (Benes et al. 2011).

Recently, attempts to estimate the degree of myocardial fibrosis in patients indicated for valve replacement were made by magnetic resonance imaging (MRI) using extracellular space volume as a proxy with the MOLLI imaging sequence (Kockova et al. 2016). The correlation of the MRI data was histologically validated using Picrosirius Red (PSR) staining on biopsies taken during surgery from the interventricular septum. Here we expand on a subset of these patients by probing the sister sections with a set of specific ECM markers working reliably on formalin-fixed, paraffin-embedded human samples, correlating them with the mass spectrometry (MS) proteomics data from another part of the biopsy cores. We hypothesized that the MS data and immunofluorescence for main fibrosis proteins will correlate with the PSR staining and MRI values. While there was a good correlation between some parameters within the single methodological approach, there is no clear link across the techniques, unless PSR staining was performed on the same tissue sections as the immunohistochemistry. Reasons for these discrepancies are discussed, leaving specific staining for ECM components in human samples still in the experimental domain.

## Materials and methods

### Patients

Patients greater than 18 years of age scheduled for aortic valve or aortic root surgery in a single institution were prospectively enrolled in our study within 1 year. All patients had a guidelines-based indication for surgical treatment (Vahanian et al. 2012; Nishimura et al. 2014). Patients with a history of myocardial infarction, previous coronary artery revascularization (other than sinus rhythm), severe renal insufficiency defined as creatinine clearance < 30 ml/min/1.72 m^2^, and a contraindication of MRI examination were excluded. The original study cohort (Kockova et al. 2016) consisted of 40 patients with an average age of 56 ± 8 years, 65% male gender. Severe aortic stenosis was present in the majority of patients (77.5%), while the remaining patients had severe aortic root dilation (7.5%) or severe aortic/mitral regurgitation (15%). However, in most cases, the valve pathology was mixed (i.e., those with stenosis presented also with an appreciable degree of regurgitant flow, and those with primary regurgitation showing a considerable transaortic pressure gradient on echocardiography; Supplemental Table 1). Since all the data necessary for cross-correlation could not be obtained from all the patients, the subset of 31 used in the present analysis (19 male, mean age 64 years, range 47–78) is provided in Supplemental Table 1. The study was approved by the Ethics Committee of Institute for Clinical and Experimental Medicine and Thomayer Hospital. Informed consent was obtained from each patient on enrollment. The study protocol conformed to the ethical guidelines of the 1975 Declaration of Helsinki.

### Myocardial sampling

The primary objective was to ensure patient safety and obtain a high-quality deep myocardial sample for analysis. The basal interventricular septum, which is easily accessible to surgeons operating on the aortic valve, was chosen for the myocardial biopsy. The biopsy was performed using a deep bioptic needle (SuperCoreTM Semi-Automatic Biopsy Instrument 16GÅ ~ 9 cm) via the left ventricular outflow tract. The obtained core (approximately 1.19 mm in diameter, length between 1–2.5 cm) divided into three parts: the first one was stored in RNAlater for future transcriptomic analysis, the second was frozen at −80 °C for MS proteomic analysis (see below), and the third one was immediately fixed in neutral buffered formalin (10%) for 24 h and processed into paraffin. The base-to-apex order of this subsampling was the same in all cores. Sections were cut in series at 5 µm, mounted on polylysine-coated slides in strips of three sections, and stained alternatively with hematoxylin/eosin and PSR as described (Kockova et al. 2016). Remaining slides were stored and stained in batches for detection of specific proteins related to myocardial fibrosis.

### Mass spectrometry (MS) quantification

All obtained samples were washed (three times) with physiological solution, heated for 7 min at 100 °C, and lyophilized. All samples (about 5 mg dry weight) were digested by trypsin as reported previously (Ost’adal et al. 2015). Samples purification, nanoliquid procedure, and tandem MS (MS/MS) analysis were done as described previously (Ost’adal et al. 2015). Database searching (Mascot score ≥ 80) was performed as described (Eckhardt et al. 2019). Sequences of peptides and M/Z used for protein identification is provided in Table 1.

**Table 1:** Details of peptide sequences and antibodies used for specific protein detection. a) M/Z and peptide sequence of proteins used in MS data; b) primary and secondary antibodies used for immunohistochemistry. M = monoclonal, P = polyclonal, Cy5 = Cyanine 5, RhRed = Rhodamine Red

a)		
Protein	M/Z	Amino acid sequence
GAPDH	865,6721	K.VIHDNFGIVEGLMTTVHAITATQK.T
GAPDH	706,4550	R.GALQNIIPASTGAAK.A
COL I	730,4399	R.GSAGPPGATGFPGAAGR.V
COL III	652,3831	R.GSPGGPGAAGFPGAR.G
Troponin I	581,9111	K.NIDALSGMEGR.K

b)			
Primary antibody	Supplier	Secondary antibody/fluorochrome	Supplier
Collagen I, M, clone COL-1, 1:500; #C2456	Sigma-Aldrich	Goat anti-Mouse, IgG/Cy5, 1:200, #115-175-146	Jackson ImmunoResearch Laboratories, Inc
Collagen III, M, clone FH-7A, 1:600; #ab6310	Abcam	Goat anti-Mouse, IgG/Cy5, 1:200, #115-175-146	Jackson ImmunoResearch Laboratories, Inc
Fibronectin, P, 1:400; #A0245	Dako	Goat anti-Rabbit/Cy5, 1:200, #111-175-144	Jackson ImmunoResearch Laboratories, Inc
Alpha Sarcomeric Actin, M, 1:800; #A2172	Sigma-Aldrich	Goat anti-Mouse, IgM/RhRed, 1:200, #115-295-075	Jackson ImmunoResearch Laboratories, Inc

### Immunohistochemistry

Alternating routine paraffin sections of 5 µm thickness were used. The slides were heat-treated twice for 5 min in citrate buffer (pH 6.0) before blocking (preincubation with 10% normal goat serum) for antigen retrieval. The antibodies used for single immunofluorescence staining were directed against collagen I (mouse monoclonal anticollagen type I, clone COL-1, 1:500; Sigma-Aldrich no. C2456) or fibronectin (rabbit polyclonal, 1:400; Dako no. A0245), and detected with cyanine 5 (Cy5)-conjugated secondary antibodies [goat anti-mouse (GaM) IgG secondary antibody Cy5-GaM, IgG, 1:200; Jackson ImmunoResearch Laboratories, Inc. no. 115–175-146 and goat anti-rabbit (GaRb) IgG secondary antibody Cy5-GaRb, 1:200; Jackson ImmunoResearch Laboratories, Inc. no. 111–175-144, respectively].

Antibody double immunofluorescence staining was performed with mouse monoclonal anticollagen type III (collagen III, clone FH-7A, 1:600; Abcam no. ab6310) and detected with Cy5-conjugated goat anti-mouse IgG secondary antibody (Cy5-GaM, IgG, 1:200; Jackson ImmunoResearch Laboratories, Inc. no. 115–175-146). Mouse monoclonal IgM isotype to alpha sarcomeric actin (SA, clone 5C5, 1:800; Sigma-Aldrich no. A2172) was used as the second primary antibody (marker of cardiomyocytes). SA antibody was detected with rhodamine red-conjugated (RhRed) goat anti-mouse IgM secondary antibody (RhRed-GaM, IgM, 1:200; Jackson ImmunoResearch Laboratories, Inc. no. 115–295-075).

All previously noted immunofluorescence antibody staining was supplemented by Alexa Fluor 488-conjugated wheat germ agglutinin lectin histochemistry (WGA, 1:50; Invitrogen no. W11261) that binds to *N*‐acetylglucosamine and *N*‐acetylneuraminic acid residues for detection of basal membranes outlining the cardiomyocytes and the overall staining of the extracellular matrix.

The nuclei were then counterstained with Hoechst 33,342 (1:100 000; Sigma-Aldrich no. 861,405). The immunofluorescently stained sections were dehydrated in ascending ethanol series, cleared in xylene, and mounted into Histokit (Carl Roth no. T160.2).

All the antibodies (Table 1) are from commercial providers and were validated previously for their specificity using various assays (western Blot, Elisa). Negative controls included incubation with nonimmune mouse or rabbit IgG and omission of the primary antibody, resulting in lack of specific immunofluorescence; for practical purposes, autofluorescence from the channel not used for labeling was used in the composite imaging.

For multiplexing purposes (Wegner et al. 2017), three additional samples were first stained with antifibronectin/WGA combination, and after imaging, they were uncoverslipped and restained with PSR. Imaging was then performed on a slide scanner as for the other sections, followed by fluorescence detection of PSR using the TRITC filter set and green laser excitation using the ×20 objective and same pixels settings as for the immunohistochemistry.

### Quantitative and semiquantitative evaluation

**Fig. 1:**
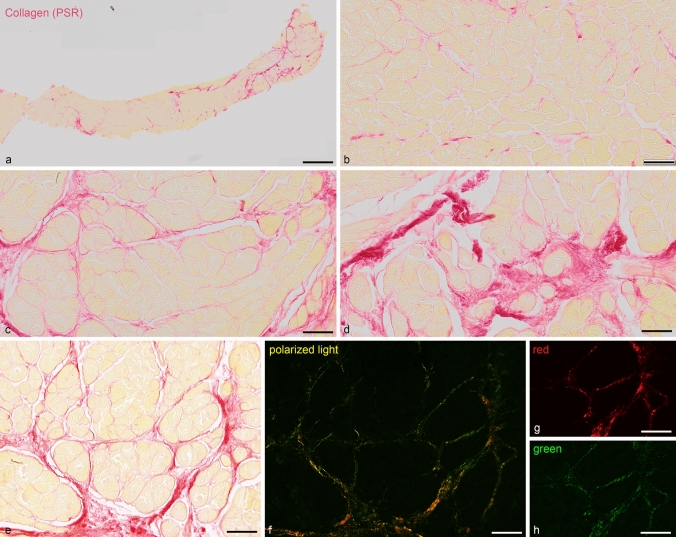
Heterogeneity within the biopsy sample. **a** panoramic scan of the biopsy core. Higher power (×20) views from this sample show areas of virtually no fibrosis (**b**), moderate perivascular fibrosis (**c**), and more severe replacement fibrosis (**d**). Panels **e** and **f** show brightfield and corresponding polarized light view of a moderately fibrotic area. Panels **g** and **h** then show a subregion from the polarized light picture split into red (**g**) and green (**h**) channels using fluorescent filter cubes, highlighting the mature and growing collagen fibers, respectively. Scale bar, 500 µm (**a**) and 50 µm (**b**–**h**)

Intensity of PSR staining was measured over the entire sample area (Fig. 1) as described (Kockova et al. 2016; Benes et al. 2011). Briefly, scans of slides performed using a ×20, NA 0.7 objective on an Olympus slide scanner fitted with an Olympus XC10 CCD camera, 1376 × 1032 pixels, 14-bit A/D conversion with shading correction, and effective pixel size of 0.323 µm were thresholded in the green channel (where the red staining of collagen was the most prominent); the area over threshold was measured. In the same field, blue channel (showing the best contrast between the sample and background) was similarly thresholded, and its area was measured in FIJI (fiji.sc). Polarized light images were taken on an Olympus BX51 upright microscope fitted with an Olympus DP80 CCD camera (24-bit depth and 1360 × 1024 pixels) through CellSens software. Percentage of PSR-positive pixels over total sample tissue area was then calculated in MS Excel. The investigators were blinded to the patient status, and two independent observers performed all the measurements, recording also the thresholds used.

For immunohistochemistry, scans of maximal possible (on average over 50% of the sample) area were made using ×20, NA 0.75 objective on an Olympus BX61 upright microscope fitted with the FluoView 1000 confocal system (Olympus, now Evident, Tokyo, Japan) using 2048 × 2048 box size (pixel dimension 0.310 microns). For more detailed resolution, ×40 oil immersion objective (NA 1.30) was used with 0.5-micron Z steps (4–6 optical sections, box 1024 × 1024 pixels, and pixel size 0.155 microns). The images were originally digitized at 12-bit depth, then converted to 8 bits per channel. The nuclear (Hoechst, excitation 405 nm), fluorescein (Alexa488-WGA, excitation 488 nm), rhodamine (tissue autofluorescence, excitation 543 nm), and far red (secondary antibody coupled with Cy5, excitation 630 nm) channels were scanned sequentially. Pattern of WGA staining was determined from the ×20 field, taking care to avoid overestimation from areas in close proximity of larger coronary vessel branches (rare). The intensity was graded from 1 (light staining of myocyte basement membrane, very thin septa between larger bundles) to 3 (large amount of intensely stained ECM among myocardial bundles, often with thickened basement membrane). Collagen III and fibronectin expression was similarly graded from 0 (not detectable), 1 (detectable in small amount in the septa), 2 (same as 1, but considerably stronger expression), to 3 (strong clusters in the thickened septa, within the WGA-positive matrix).

**Fig. 2:**
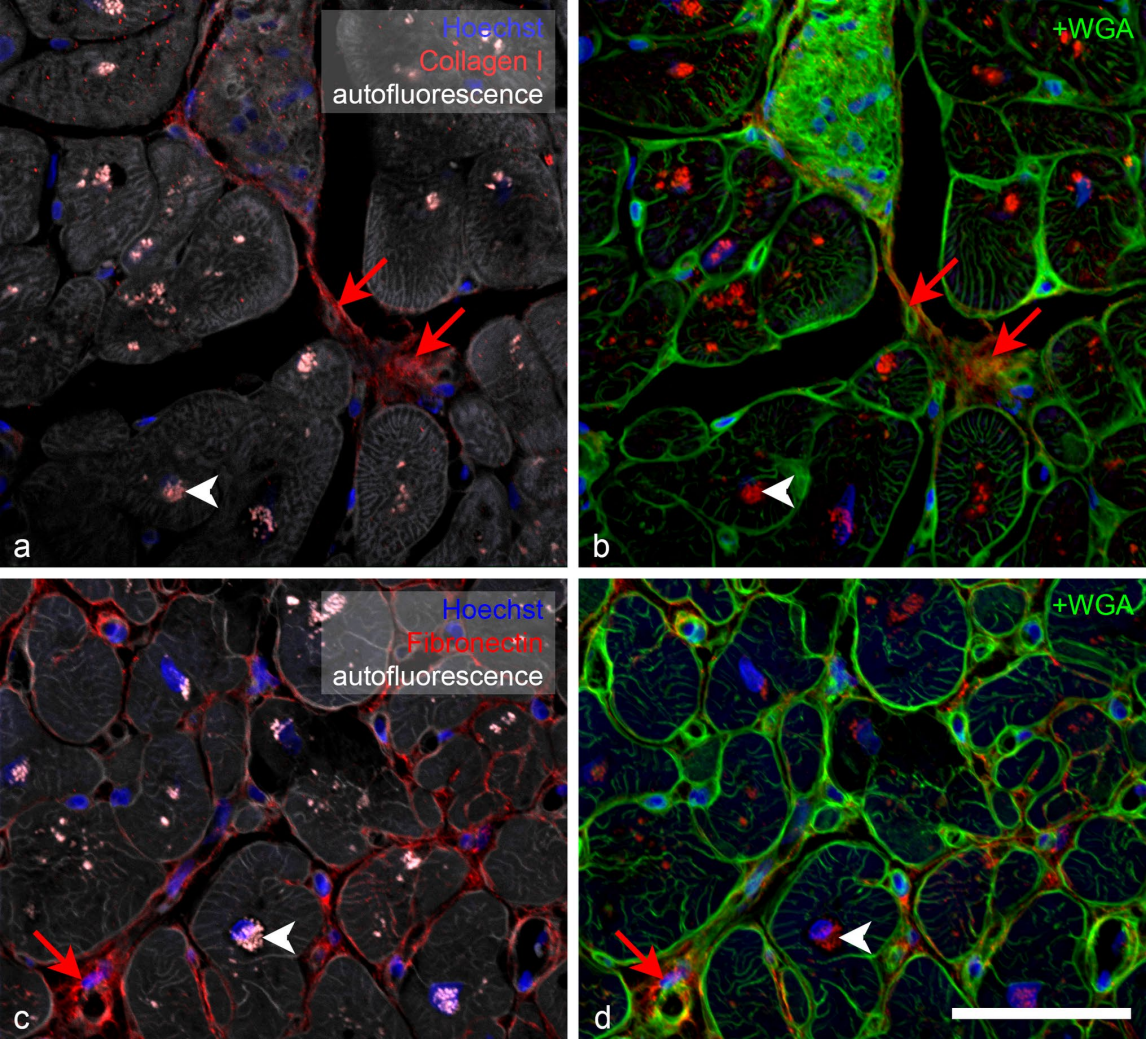
High power confocal views (×40 oil) of collagen I and fibronectin immunostaining demonstrating signal specificity. Antibody signal is in the red channel (red arrows), and the left panels show tissue autofluorescence from the rhodamine channel (grey). Bright autofluorescence is present inside the cells in the perinuclear area (bright white dots and white arrowheads). The right set of panels shows the antibody staining in the red channel, together with nuclear (blue) and WGA (green) signal. Specific antibody binding (arrows) occurs in the extracellular matrix, in contrast to strongly autofluorescent lipofuscin granules (arrowheads) located in the cytoplasm. Scale bar, 100 µm

**Fig. 3:**
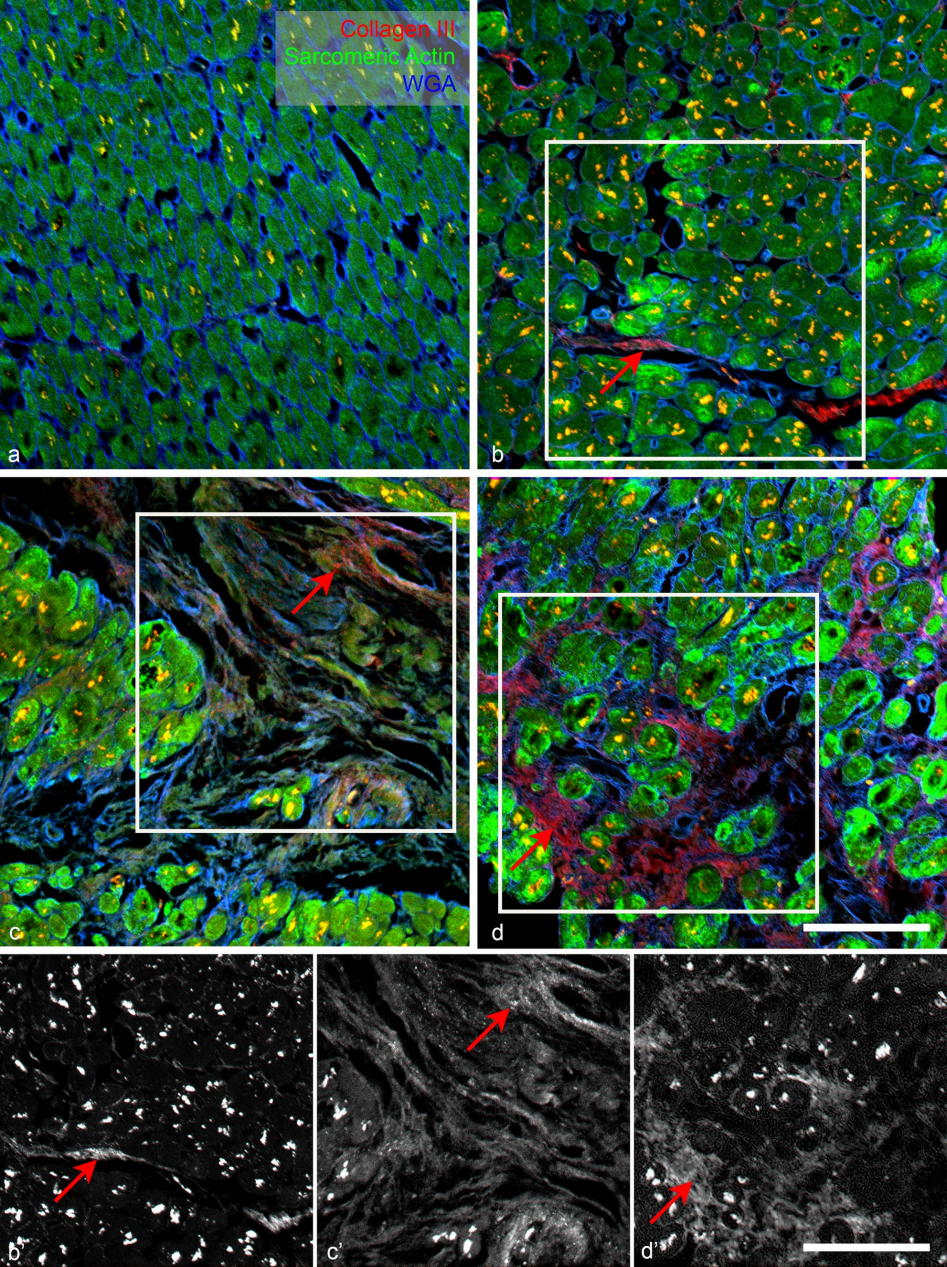
Examples of different levels of immunofluorescent staining extent and intensity in four different patient samples: collagen III (red). Grade 0 (no detectable staining) **a**; grade 1 (small amount of staining in thin septa between myocyte bundles) **b**; grade 2 (more intense staining within fibrotic areas) **c**; grade 3 (intense staining in most of the WGA-positive blue areas) **d**. Green channel shows myocytes stained with sarcomeric actin antibody. ×20 objective, single confocal sections; scale bar, 100 µm

**Fig. 4:**
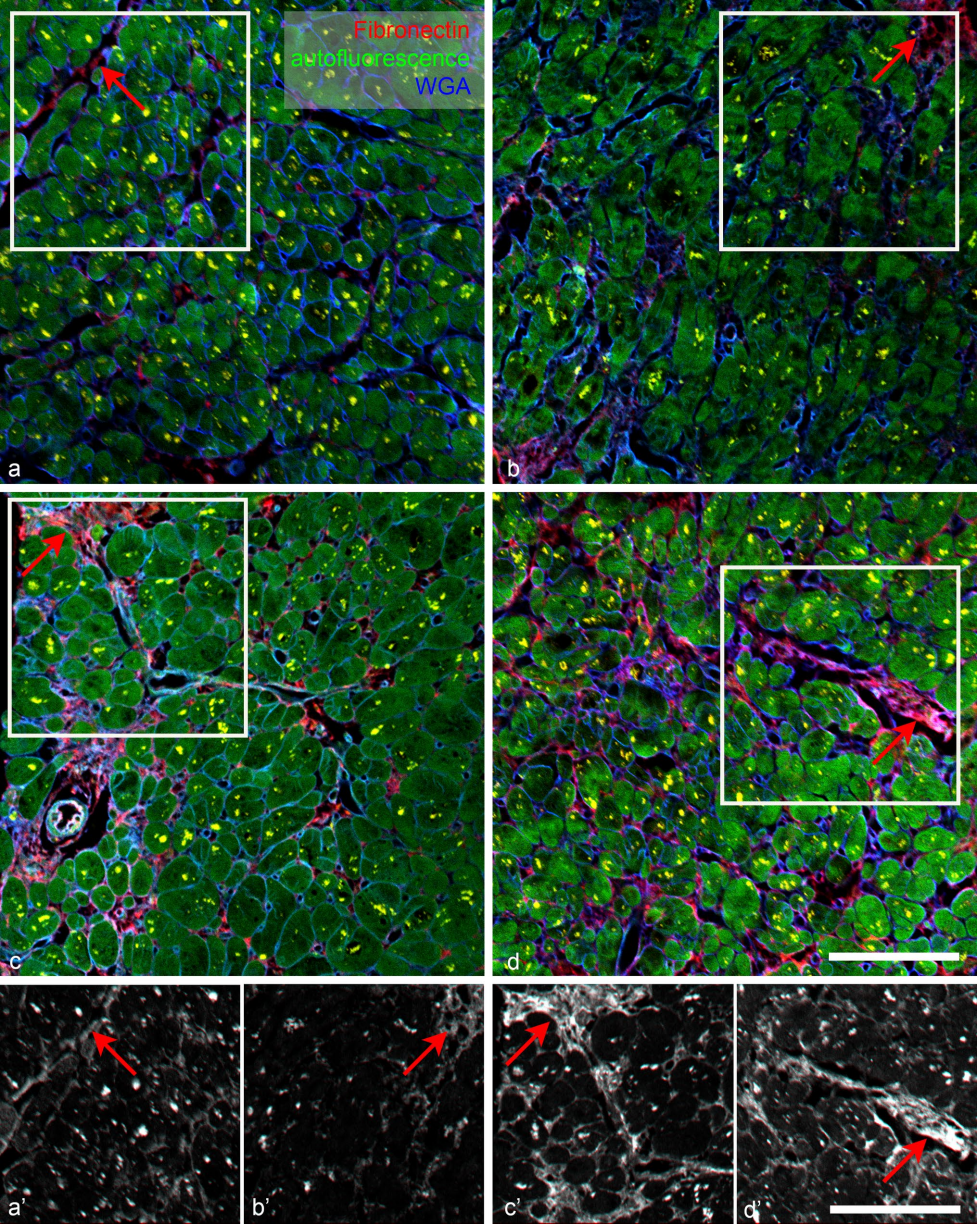
Examples of different levels of immunofluorescent intensity in four different patient samples: fibronectin (red). Grade 1 (only faint detectable specific staining) **a**; grade 2 (small patches in fibrotic areas between myocyte bundles, **b**; grade 3 (intense staining within the fibrotic areas), **c**, **d**. Green channel shows myocyte autofluorescence and WGA staining (cell borders and ECM) is in blue; red arrows point to specific staining in the ECM. Bright lipofuscin granules inside myocytes appear yellow. ×20 objective, single confocal sections; scale bar, 100 µm

For presentation, the images were assembled and labeled in Adobe Photoshop. Digital image manipulations included adjustment of levels (separately for each channel), Unsharp Mask filtering (performed only in Fig. 1 for presentation purposes, not on images for quantification), and channel switching to enhance clarity of the Figs. 2–4. All transformations were performed on the entire image.

### Correlations and statistics

Tabular data were processed in MS Excel. Correlations among different markers are expressed as scatter plots. Where appropriate, linear regression line and equation is fitted and displayed. Correlation coefficient > 0.2 was considered as biologically relevant.

## Results and discussion

Most samples examined showed fair amount of local inhomogeneities in the contents of collagen visualized by PSR (Fig. 1). For quantification, unbiased selection or, better yet, systematic sampling is advisable; as otherwise, a wide range of values can be easily obtained (see examples in Fig. 1). It was found that using polarized light, graphic results illustrating either high amount of fibrosis or its lack could be readily obtained (Papousek et al. 2020), but it was unsuitable for quantification for several reasons: (1) problem with background signal, as tissue edges as well as sarcomeric proteins also show up in polarized light; (2) uneven field illumination, as the camera’s shading correction did not function well in this mode, making thresholding difficult; (3) problems setting correct exposure time, as there were large differences in pixel brightness, resulting often in overexposure of bright pixels while simultaneously missing fine fibers, even at ×20 magnification with pixel size of 0.32 microns. Using fluorescent filter cubes, we were able to separate the green and red signal but did not pursue this any further, since most hearts with an appreciable degree of fibrosis showed good signal in both channels (Fig. 1).

WGA staining showed visually similar staining pattern as the PSR, labeling all the fibrillar extracellular matrix and outlining the myocyte borders and capillaries. It was more extensive (Figs. 2, 3, 4) than PSR, which labeled predominantly the wavy collagen fibers. When combined with the sarcomeric actin staining, it showed neatly the areas of replacement fibrosis (Fig. 3). WGA is thus an excellent counterstain for myocardial samples, allowing cell diameter measurements and highlighting the areas of abundant ECM presence.

Strong lipofuscin autofluorescence from granules in the perinuclear region was visible in all channels, complicating interpretation of antibody staining. Combination of myocyte marker (Fig. 3) or autofluorescence (Fig. 4) and ECM marker WGA (Figs. 3, 4) allowed us to distinguish the specific staining in the ECM from lipofuscin autofluorescence. There was no difference in the lipofuscin intensity signal between the autofluorescence, negative control, and section stained with the antibody in the same (far red, Cy5) channel.

**Fig. 5:**
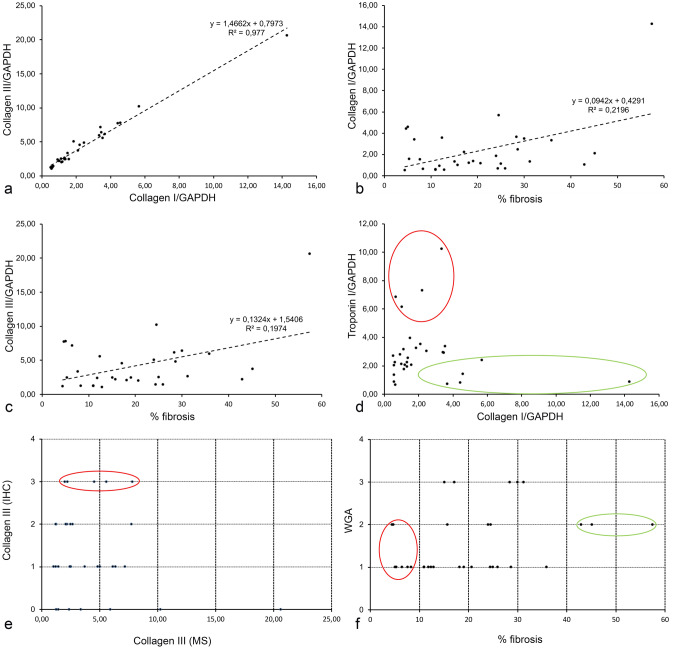
Cross-correlation of different fibromarkers. **a** Good agreement between amounts of collagen I and III detected by MS. **b** Loose correlation between amount of collagen III detected by MS and percentage of sample fibrosis measured by PSR. **c** Loose correlation between amount of collagen I detected by MS and percentage of sample fibrosis measured by PSR. **d** Inverse relationship between a cardiomyocyte marker (troponin) and a fibromarker (collagen I) expression detected by MS. The highest troponin values (red ellipse) show low collagen I numbers, and vice versa (green ellipse). **e** Lack of correlation between semiquantitative collagen III detected by immunohistochemistry and quantitative MS data. The relatively highest relative expression (red ellipse) is found in samples with moderate MS values. **f** Relationship between semiquantitative fibrillar ECM detected by WGA and percentage of fibrosis measured by PSR. There is no clear correlation between these two markers

Collagen III immunofluorescence staining was detectable in a majority of samples (24 of 31, Supplemental Table 1). When present, the staining was embedded within the WGA-positive ECM or located within the thickened septa (Fig. 3), corresponding with the PSR staining pattern. Collagen III is a major structural component in hollow organs such as the heart, as well as in many other tissues. It is associated with collagen I. We chose to stain the sections with collagen III because of the limited number of slides available for parallel staining and because the signal with this antibody was better than that of collagen I. Furthermore, both collagen I and collagen III were shown to be increased in cardiac fibrosis (Schimmel et al. 2022), as was confirmed also by our proteomic analysis (Fig. 5). Collagen III is more elastic than Collagen I (Cleutjens et al. 1995) and change of their ratio in favor of Collagen I, which has a higher cross-linking potential increasing myocardial stiffness was reported in fibrosis progression (Rain et al. 2016).

Positive fibronectin immunofluorescence staining was also present in a majority of samples (29 of 31). The staining pattern was slightly more widespread than that of collagen III and was often present in the gaps between the individual cardiomyocytes (Fig. 4). Interestingly, its patchy expression was distinct from the fibrillar WGA staining, evidenced by little colocalization. However, it was associated with the perivascular fibrosis and thicker septa between myocardial bundles. Fibronectin is found in two forms, soluble plasma fibronectin produced mainly by the liver and insoluble ECM component produced mainly by the fibroblasts. Fibronectin is a high molecular weight glycoprotein, which mediates cell adhesion through integrins. It also binds to numerous other ECM proteins, including collagens. Changes in fibronectin expression, organization, and turnover were reported under numerous pathological conditions including cancer and fibrosis, and inhibition of its expression or polymerization attenuated adverse remodeling in a mouse model of postischemic fibrosis (Valiente-Alandi et al. 2018).

Fluorescent detection of proteins of interest within the myocardium is not completely straightforward due to considerable background fluorescence due mainly to conjugated double bonds found in myoglobin. This background could be even used for label-free tissue imaging as reported previously (Buffinton et al. 2013), as well as more recently for specific detection of proteins of interest using second harmonic generation (Hadraba et al. 2017). In the aged and diseased heart, there is an additional false-positive signal from lipofuscin, which emits strongly at all wavelengths (Kakimoto et al. 2019). Fortunately, for detection of ECM molecules, it is not an issue, since its localization is strictly intracellular around the nucleus (Fig. 2); if desired, the specific antibody signal could be obtained by subtraction of the autofluorescence channel.

A total of 107 proteins were identified with the MS/MS approach, and the list is summarized in Supplemental Table 2. It includes predominantly high-abundance structural proteins, such as sarcomeric components (actin, myosin, troponin), extracellular matrix components (collagens), and common metabolic enzymes (GAPDH, CK-M, and cytochrome oxidases).

To enable comparison among samples, abundance of proteins detected by specific peptide fragments was normalized to GAPDH. As expected, excellent correlation (R^2^ > 0.9) was found between relative levels of collagen I and collagen III (Fig. 5). There was a weak inverse correlation between sarcomeric protein (troponin I) expression and collagen I. However, the correlation between the MS and immunohistochemistry or PSR data was generally weak. We can offer several explanations for this discrepancy. First, the fibrosis within the myocardium is quite heterogeneous (Fig. 1). Since the MS and histological analysis were performed on different portions of the biopsy core, it is quite possible that these two samples would show differences, even if analyzed with the same method. There are numerous gradients within the heart, such as myocyte diameter across the wall (Clubb et al. 1987) or in protein composition along the apex-to-base axis (Eckhardt et al. 2018); it is thus likely that the biopsy core, sampled longitudinally from the interventricular septum (on which little information is available), will show similar gradients. Second, the technique for MS measurements involves trypsin digestion. This is dependent on the degree of collagen cross-linking (Knitlova et al. 2021), which is likely higher in cases of severe fibrosis (Lopez et al. 2012). Thus, the amount of collagen in such samples could be systematically underestimated by the MS. Third, the semiquantitative grading system is not as precise as the quantification provided by either PSR or MS; we excluded the samples where no values were obtained on MS but kept those where tissue was clearly present, but the fibronectin or collagen III was not detected (grade 0). There was not a clear correlation even between WGA and PSR staining; this could be due to differences within the sample, as the sections were about 10–20 µm apart.

The lack of staining correlation on microscale could be explained by the fact that PSR was quantified from the entire section, while the immunohistochemistry was evaluated from a smaller subset of it (about 25–50% of the section area). As can be appreciated from Fig. 1, there is a fair amount of variation even within the whole sectioned core, so unless one chooses the same area for all measurements, the correlation is not perfect. We measured PSR staining in as large area as possible to correlate it primarily with the MRI data (Kockova et al. 2016), which are from even larger voxels (2 × 2 mm × 8 mm). However, collagen or fibronectin fluorescent immunostaining required much higher magnification, so it was impractical to scan systematically the entire field, not to mention the heterogeneities around larger vessels that were systematically excluded, as is the custom in evaluation of myocardial fibrosis.

**Fig. 6:**
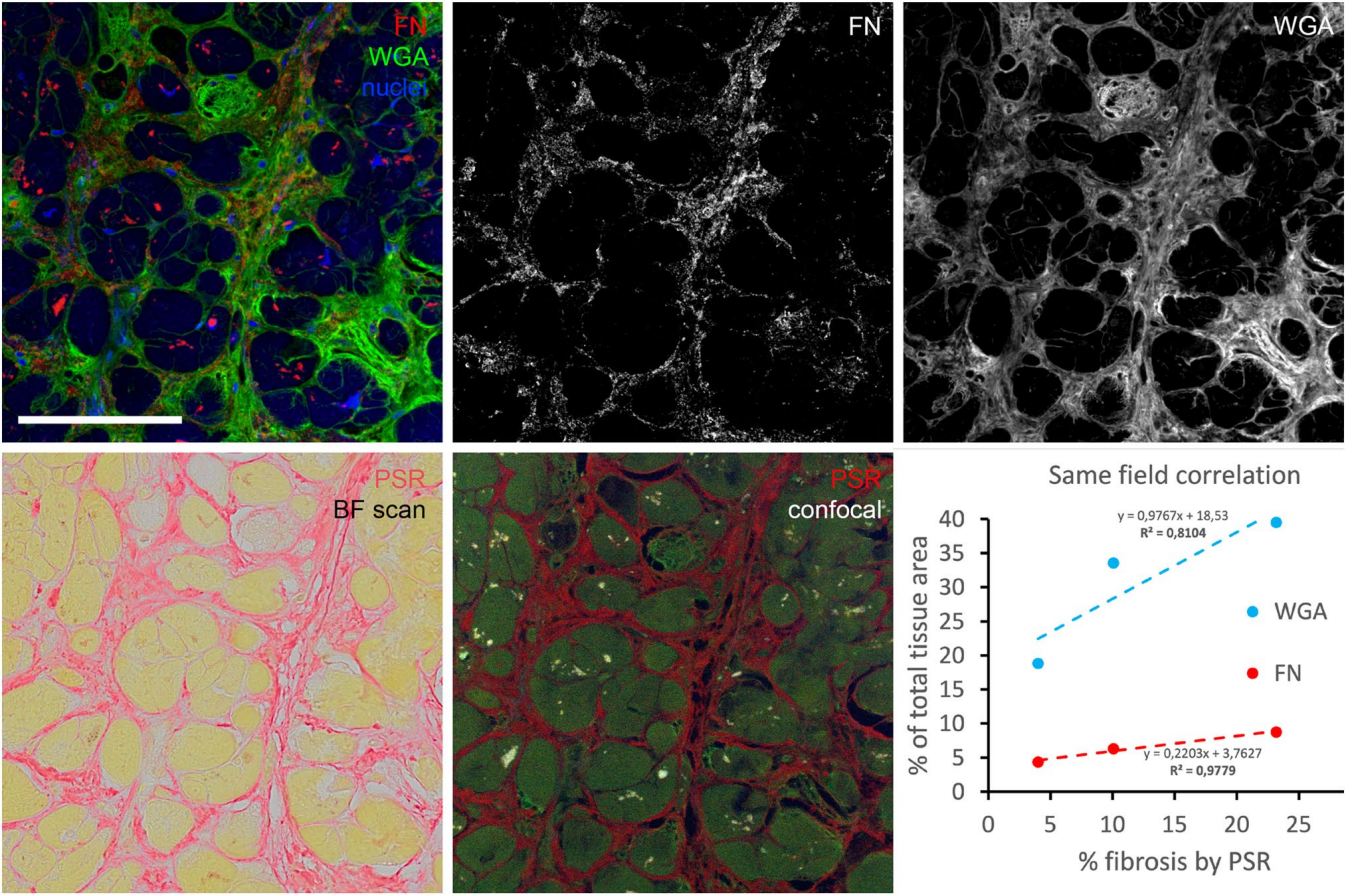
Multiplexing of fibronectin (FN) immunohistochemistry with Picrosirius Red (PSR) staining. Top panels show an area immunolabeled with FN antibody (red), wheat germ agglutinin (WGA, green), and DAPI (blue). Extracted single-channel pictures (FN masked to exclude autofluorescent lipofuscin intracellular granules) used for thresholding and quantification are also shown. Bottom row shows exactly the same area stained afterwards with PSR. The first panel shows the brightfield scan acquired with ×20 objective (used consistently for quantifications); the fluorescence image was obtained as suggested (Wegner et al. 2017) on confocal microscope using the same magnification as above, and the correlation plot was obtained by this type of analysis using three of the samples with various amount of fibrosis. WGA labels more extensive area then PSR, which is specific to collagens (even after antigen retrieval protocol, which increases the positive area as reported (Gadd 2014)). In contrast, the FN-positive area is a subset of the WGA-positive region. There is good correlation among these markers if used on the same region of interest. Scale bar, 100 µm (identical for all panels)

To resolve this discrepancy, we performed PSR staining on samples stained prior to it by the fibronectin/WGA combination and quantified the proportion of staining positivity in three samples (leftover unstained slides from three samples with different fibrosis levels). As can be appreciated in the Fig. 6, there is a decent correlation between the percentage of PSR positivity, WGA staining (more abundant due to simultaneous detection of basement membranes and capillaries), and fibronectin immunopositivity (more restricted within the WGA-positive areas) when measured in the identical region of interest. Such multiplexing approach was described by Wegner and colleagues (Wegner et al. 2017) but performed in the opposite order (i.e., the PSR staining first followed by destaining in the antigen retrieval step), as heating during the antigen retrieval process results in degradation of fine collagen architecture and increases the area of PSR immunopositivity (Gadd 2014). Here we show that good correlation could be obtained in both ways (i.e., PSR first or immunofluorescence first), which is a useful tip for those wishing to reanalyze already stained or archival sections for comparative/correlative purposes.

It was disappointing that, contrary to our hypothesis and results from animal studies, we could not correlate the data (notably the percentage of fibrosis) with measurable clinical parameters, such as left ventricular pressure gradient (TAPG, Supplemental Table 1). When the patients were grouped according to its severity, we found, nevertheless, two of the “top five” fibrotic samples in the bottom five patients according to the pressure gradient; conversely, two of the five with the lowest fibrosis levels (below 6%, i.e. considered a normal finding) were in the top five patients with the highest TAPG.

In summary of the main study limitations, while we had access to ventricular samples from a well-defined region, the septum might not respond to pressure/volume overload in the same way as the left ventricular free wall. Thus, careful analysis of a well-defined animal model should uncover correlation between the septal and left ventricular wall values. Another problem was that the biopsy core had to be divided for histological and biochemical analysis, so the proteomic data come from an adjacent, but not identical, region of the myocardium, which could be a problem with large regional variations in some parameters. Third, unlike in animal studies where a well-defined experimental (and control) group of typically young and otherwise healthy animals of single sex is used, our sample represents a heterogeneous group with various comorbidities, which, at this sample size, may complicate a clear-cut interpretation.

This analysis shows that ECM proteins involved in cardiac fibrosis—collagen I, collagen III, and fibronectin—can be detected in routine human ventricular myocardium biopsies by immunofluorescence and separated from strong endogenous tissue autofluorescence. However, their relative amounts show little correlation with the gold standard for fibrosis quantification (PSR) or with collagen quantity detected by MS, most likely due to high variability at the microscale level. Thus, we can not recommend any of those as markers for routine use in clinical practice; more experimental studies using well-defined models are needed to resolve this issue. For correlation studies of expression of two proteins, double immunofluorescence on a single section should be used whenever possible; if not, immediately adjacent ones are most likely to yield the most informative results. Alternatively, multiplexing immunohistochemistry with PSR staining, albeit more cumbersome than using sister sections, would yield the most precise data and worked well on our samples.

### Supplementary Information

Below is the link to the electronic supplementary material.

Supplementary file1 Supplementary Table 1 List of patients whose biopsies were used in this study with their characteristics. In the first page, the patients are grouped by the amount of fibrosis histologically detected by PSR, with the top 5 values highlighted in red, and bottom 5 in green. The second page shows the same data sorted according to the transaortic pressure gradient (PDF 154 KB)

Supplementary file2 Supplementary Table 2 List of proteins detected in human ventricular biopsy samples by MS/MS (PDF 39 KB)

## Data Availability

All primary datasets are available from the corresponding author upon reasonable request.
