# Supplementary material for: Fibrosis and expression of extracellular matrix proteins in human interventricular septum in aortic valve stenosis and regurgitation
Source: Histochem Cell Biol. 2024 Feb 12;161(5):367–79. doi: 10.1007/s00418-024-02268-y (PMC11045568; doi:10.1007/s00418-024-02268-y)
Supplement: Supplementary file 1 — Supplementary file1 Supplementary Table 1 List of patients whose biopsies were used in this study with their characteristics. In the first page, the patients are grouped by the amount of fibrosis histologically detected by PSR, with the top 5 values highlighted in red, and bottom 5 in green. The second page shows the same data sorted according to the transaortic pressure gradient (PDF 154 KB) [file 418_2024_2268_MOESM1_ESM.pdf]

Patient no.	Age	Sex	Main Dg.	TAPG	AR grade (0-4)	Comorbidities	*% fibrosis PSR*	*Coll 1/GAPDH*	*Coll 3/GAPDH*	Tropomyosin	WGA IHC	Coll 3 IHC	FN IHC	*myocyte appearance*
1	78	M	AS	37	1	PAD, HT, IHD	57,5	14,28	20,63	0,86	2	0	3	uneven
2	58	M	AS	35	2	HT, IHD	45,2	2,09	3,72	3,52	2	1	3	normal
3	63	F	ASC AA	?	2	-	43,0	1,03	2,19	6,15	2	2	2	normal
4	54	F	ASC AA	?	2	-	35,9	3,33	5,92	10,23	1	0	0	normal
5	68	M	AS/AR	72	2.5	HT	31,2	1,33	2,66	1,95	3	2	1	normal
6	64	M	AS	54	2	PAD, HT	30,0	3,47	6,37	2,90	3	1	2	uneven
7	47	M	AR	25	4	-	28,6	2,46	4,83	3,03	1	1	0	normal
8	64	M	AS	55	1		28,4	3,66	6,12	0,73	3	1	3	uneven
9	70	F	AS	60	0	HT	26,0	0,65	1,43	0,65	1	1	1	poorly stained, uneven
10	57	F	AS	53	1	HT	25,0	1,11	2,52	3,16	1	1	1	normal
11	76	M	AS	69	0	-	24,5	5,67	10,23	2,40	2	0	2	normal
12	69	F	AS	46	0	HT	24,4	0,68	1,44	6,84	1	0	2	normal
13	51	M	AR	?	4	HT, IHD	24,0	1,85	5,03	3,26	2	1	2	normal
14	74	F	AR	44	3	HT	20,7	1,14	2,01	1,77	1	3	2	normal
15	62	F	AS	110	1	HT	19,1	1,37	2,45	2,55	1	1	3	hypertrophic
16	53	M	AS	54	1	HT	18,2	1,20	2,07	2,05	1	2	3	normal
17	49	M	AR	?	3	HT	17,2	2,21	4,53	7,30	3	3	2	uneven]
18	69	F	AS	47	1	PAD, HT	15,8	0,99	2,21	2,11	2	3	1	normal
19	56	M	AR	45	4	-	15,1	1,32	2,47	2,25	3	2	2	normal
20	67	M	AS	51	1	PAD, HT	12,9	0,57	1,03	1,35	1	1	1	normal
21	67	F	AS	45	2	DIET, HT	12,4	3,54	5,57	3,37	1	3	3	normal
22	72	F	AS	66	2	HT	11,8	0,92	2,36	2,79	1	0	1	normal
23	68	M	AS	?	0	HT, IHD	11,0	0,58	1,22	2,02	1	1	2	uneven
24	62	M	AS	72	1	-	11,0	0,56	1,26	0,88	1	0	3	normal
25	75	M	ASC AA	?	2	HT	8,3	0,63	1,24	2,24	1	2	1	normal
26	75	M	AS	42	1	-	7,6	1,51	3,36	3,95	1	0	3	normal
27	57	M	AS	37	0	HT, IHD	6,4	3,42	7,18	2,93	1	1	2	normal
28	69	F	AS	93	1	DIET, HT	5,3	1,57	2,45	2,05	1	1	1	normal
29	61	M	AS	83	0	IHD	5,1	4,57	7,80	1,41	1	3	3	normal
30	66	M	AS	52	2	HT, IHD	4,7	4,43	7,73	0,82	2	2	2	normal
31	59	F	AS	22	1	HT, IHD	4,4	0,53	1,21	2,71	2	2	1	poorly stained
**mean**	**64**													
**range**	**47 - 78**													

31 patients subgroup, 19 male

Ranged by % fibrosis by Picrosirius Red staining. Top 5 and bottom 5 values highlighted in red and green, respectively.

Abbreviatio

AS	aortic stenosis
AR	aortic regurgitation
PAD	diabetes on peroral antidiabetic drugs
HT	arterial hypertension
IHD	ischemic heart disease
WGA	wheart germ agglutinin
IHC	immunohistochemistry
DIET	diabetes type 2 on diet
ASC AA	ascending aorta aneurysm
TAPG	transaortic mean pressure gradient (mm Hg)
Coll	collagen
FN	fibronectin

Patient no.	Age	Sex	Main Dg.	TAPG	AR grade (0-4)	Comorbidities	*% fibrosis PSR*	*Coll 1/GAPDH*	*Coll 3/GAPDH*	Tropomyosin	WGA IHC	Coll 3 IHC	FN IHC	*myocyte appearance*
31	59	F	AS	22	1	HT, IHD	4,4	0,53	1,21	2,71	2	2	1	poorly stained
7	47	M	AR	25	4	-	28,6	2,46	4,83	3,03	1	1	0	normal
2	58	M	AS	35	2	HT, IHD	45,2	2,09	3,72	3,52	2	1	3	normal
1	78	M	AS	37	1	PAD, HT, IHD	57,5	14,28	20,63	0,86	2	0	3	uneven
27	57	M	AS	37	0	HT, IHD	6,4	3,42	7,18	2,93	1	1	2	normal
26	75	M	AS	42	1	-	7,6	1,51	3,36	3,95	1	0	3	normal
14	74	F	AR	44	3	HT	20,7	1,14	2,01	1,77	1	3	2	normal
19	56	M	AR	45	4	-	15,1	1,32	2,47	2,25	3	2	2	normal
21	67	F	AS	45	2	DIET, HT	12,4	3,54	5,57	3,37	1	3	3	normal
12	69	F	AS	46	0	HT	24,4	0,68	1,44	6,84	1	0	2	normal
18	69	F	AS	47	1	PAD, HT	15,8	0,99	2,21	2,11	2	3	1	normal
20	67	M	AS	51	1	PAD, HT	12,9	0,57	1,03	1,35	1	1	1	normal
30	66	M	AS	52	2	HT, IHD	4,7	4,43	7,73	0,82	2	2	2	normal
10	57	F	AS	53	1	HT	25,0	1,11	2,52	3,16	1	1	1	normal
6	64	M	AS	54	2	PAD, HT	30,0	3,47	6,37	2,90	3	1	2	uneven
16	53	M	AS	54	1	HT	18,2	1,20	2,07	2,05	1	2	3	normal
8	64	M	AS	55	1		28,4	3,66	6,12	0,73	3	1	3	uneven
9	70	F	AS	60	0	HT	26,0	0,65	1,43	0,65	1	1	1	poorly stained, uneven
22	72	F	AS	66	2	HT	11,8	0,92	2,36	2,79	1	0	1	normal
11	76	M	AS	69	0	-	24,5	5,67	10,23	2,40	2	0	2	normal
5	68	M	AS/AR	72	2.5	HT	31,2	1,33	2,66	1,95	3	2	1	normal
24	62	M	AS	72	1	-	11,0	0,56	1,26	0,88	1	0	3	normal
29	61	M	AS	83	0	IHD	5,1	4,57	7,80	1,41	1	3	3	normal
28	69	F	AS	93	1	DIET, HT	5,3	1,57	2,45	2,05	1	1	1	normal
15	62	F	AS	110	1	HT	19,1	1,37	2,45	2,55	1	1	3	hypertrophic
3	63	F	ASC AA	?	2	-	43,0	1,03	2,19	6,15	2	2	2	normal
4	54	F	ASC AA	?	2	-	35,9	3,33	5,92	10,23	1	0	0	normal
13	51	M	AR	?	4	HT, IHD	24,0	1,85	5,03	3,26	2	1	2	normal
17	49	M	AR	?	3	HT	17,2	2,21	4,53	7,30	3	3	2	uneven]
23	68	M	AS	?	0	HT, IHD	11,0	0,58	1,22	2,02	1	1	2	uneven
25	75	M	ASC AA	?	2	HT	8,3	0,63	1,24	2,24	1	2	1	normal

mean **64**

range **47 - 78**

Sorted by TAPG, low to high

31 patients subgroup, 19 male

Ranged by % fibrosis by Picrosirius Red staining. Top 5 and bottom 5 values highlighted in red and green, respectively.

Abbreviatio AS aortic stenosis

Abbreviatio	AS	aortic stenosis
	AR	aortic regurgitation
	PAD	diabetes on peroral antidiabetic drugs
	HT	arterial hypertension
	IHD	ischemic heart disease
	WGA	wheart germ agglutinin
	IHC	immunohistochemistry
	DIET	diabetes type 2 on diet
	ASC AA	ascending aorta aneurysm
	TAPG	transaortic mean pressure gradient (mm Hg)
	Coll	collagen
	FN	fibronectin
