# Supplementary material for: Fibrosis and expression of extracellular matrix proteins in human interventricular septum in aortic valve stenosis and regurgitation
Source: Histochem Cell Biol. 2024 Feb 12;161(5):367–79. doi: 10.1007/s00418-024-02268-y (PMC11045568; doi:10.1007/s00418-024-02268-y)
Supplement: Supplementary file 2 — Supplementary file2 Supplementary Table 2 List of proteins detected in human ventricular biopsy samples by MS/MS (PDF 39 KB) [file 418_2024_2268_MOESM2_ESM.pdf]

No.	Accession n.	Protein name	MW [kDa]	Scores	SC [%]
1	IPI00025880	MYH7 Myosin-7	223.0	8927.9	53.8
2	IPI00514201	MYH6 Myosin-6	223.6	6150.8	36.2
3	IPI00297646	COL1A1 Collagen alpha-1(I) chain	138.9	2801.8	39.4
4	IPI00759754	TTN Isoform 1 of Titin	3813.8	2364.6	1.7
5	IPI00019884	ACTN2 Alpha-actinin-2	103.8	1979.3	42.2
6	IPI00021033	COL3A1 Isoform 1 of Collagen alpha-1(III) chain	138.5	1714.8	22.0
7	IPI00303476	ATP5B ATP synthase subunit beta, mitochondrial	56.5	1609.8	53.3
8	IPI00465084	DES Desmin	53.5	1556.4	56.4
9	IPI00304962	COL1A2 Collagen alpha-2(I) chain	129.2	1434.6	24.3
10	IPI00023006	ACTC1 Actin, alpha cardiac muscle 1	42.0	1375.0	48.3
11	IPI00292412	MYBPC3 myosin-binding protein C, cardiac-type	140.7	1362.5	19.9
12	IPI00021428	ACTA1 Actin, alpha skeletal muscle	42.0	1305.8	5.8
13	IPI00243742	MYL3 Myosin light chain 3	21.9	1034.3	62.6
14	IPI00014581	TPM1 Isoform 1 of Tropomyosin alpha-1 chain	32.7	957.9	43.0
15	IPI00216798	MYL2 Myosin regulatory light chain 2, ventricular/cardiac muscle isoform	18.8	950.3	75.9
16	IPI00440493	ATP5A1 ATP synthase subunit alpha, mitochondrial	59.7	894.8	31.5
17	IPI01011344	ACTG1 Uncharacterized protein	37.4	827.5	5.4
18	IPI00015141	CKMT2 Creatine kinase S-type, mitochondrial	47.5	809.0	30.1
19	IPI00005587	MYOM2 Myomesin-2	164.8	708.2	10.9
20	IPI01015355	ACO2 Uncharacterized protein	83.4	659.4	18.7
21	IPI00027487	CKM Creatine kinase M-type	43.1	593.8	28.9
22	IPI00031522	HADHA Trifunctional enzyme subunit alpha, mitochondrial	82.9	551.6	21.5
23	IPI01011661	TNNT2 Uncharacterized protein	34.0	540.5	33.2
24	IPI00470359	TNNC1 Troponin C, slow skeletal and cardiac muscles	18.4	440.5	43.5
25	IPI00418471	VIM Vimentin	53.6	438.0	6.2
26	IPI00337541	NNT NAD(P) transhydrogenase, mitochondrial	113.8	409.9	10.8
27	IPI00219018	GAPDH Glyceraldehyde-3-phosphate dehydrogenase	36.0	408.7	29.9
28	IPI00305383	UQCRC2 Cytochrome b-c1 complex subunit 2, mitochondrial	48.4	381.0	18.8
29	IPI00946286	COL6A3 collagen alpha-3(VI) chain isoform 4 precursor	278.0	372.4	5.2
30	IPI00013991	TPM2 Isoform 1 of Tropomyosin beta chain	32.8	369.7	9.2
31	IPI00244346	TNNI3 Troponin I, cardiac muscle	24.0	364.7	32.9
32	IPI00298933	CASQ2 Calsequestrin-2	46.4	358.0	24.8
33	IPI00022891	SLC25A4 ADP/ATP translocase 1	33.0	345.8	27.9
34	IPI00747443	ATP2A2 Isoform 5 of Sarcoplasmic/endoplasmic reticulum calcium ATPase 2	109.7	344.8	6.9
35	IPI00413958	FLNC Isoform 2 of Filamin-C	287.1	333.7	3.8
36	IPI00434580	MYOM1 Isoform 1 of Myomesin-1	187.5	327.3	4.2
37	IPI00387020	MYOZ2 Myozenin-2	29.9	311.0	26.5
38	IPI00514458	LDB3 Isoform 1 of LIM domain-binding protein 3	77.1	300.6	7.4
39	IPI01015385	IDH2 Isocitrate dehydrogenase	45.2	279.1	16.0
40	IPI00006579	COX4I1 Cytochrome c oxidase subunit 4 isoform 1, mitochondrial	19.6	262.0	33.1
41	IPI00003482	DECR1 2,4-dienoyl-CoA reductase, mitochondrial	36.0	248.2	17.0
42	IPI00003925	PDHB Isoform 1 of Pyruvate dehydrogenase E1 component subunit beta, mitochondrial	39.2	246.8	17.3
43	IPI00291136	COL6A1 Collagen alpha-1(VI) chain	108.5	238.7	4.9
44	IPI00220416	UQCRB Cytochrome b-c1 complex subunit 7	13.5	236.8	50.5
45	IPI00910262	POSTN Isoform 4 of Periostin	83.8	223.1	9.7
46	IPI00013847	UQCRC1 Cytochrome b-c1 complex subunit 1, mitochondrial	52.6	219.8	11.5
47	IPI00098902	OGDH 2-oxoglutarate dehydrogenase, mitochondrial	115.9	218.5	6.8
48	IPI00910267	GOT2 Aspartate aminotransferase	43.0	217.7	12.1
49	IPI00478410	ATP5C1 Isoform Liver of ATP synthase subunit gamma, mitochondrial	33.0	213.7	21.5
50	IPI00178744	ACADVL Isoform 2 of Very long-chain specific acyl-CoA dehydrogenase, mitochondrial	68.0	211.6	4.6
51	IPI01011882	DLD cDNA FLJ50515, highly similar to Dihydrolipoyl dehydrogenase, mitochondrial	43.6	208.3	11.2
52	IPI00294398	HADH Isoform 1 of Hydroxyacyl-coenzyme A dehydrogenase, mitochondrial	34.3	208.0	14.0
53	IPI00220487	ATP5H Isoform 1 of ATP synthase subunit d, mitochondrial	18.5	207.9	34.2

54	IPI00917575	HSPD1 cDNA FLJ51046, highly similar to 60 kDa heat shock protein, mitochondrial	55.0	206.2	11.8
55	IPI00383539	CS Citrate synthase	50.4	206.1	12.4
56	IPI00878623	MB Uncharacterized protein	16.0	202.0	25.2
57	IPI00917605	CYCS Uncharacterized protein	11.3	197.3	17.8
58	IPI00017510	MT-CO2 Cytochrome c oxidase subunit 2	25.5	185.8	16.3
59	IPI00384992	MYL4 Myosin light chain 4	21.6	182.4	14.2
60	IPI00026964	UQCRFS1 Cytochrome b-c1 complex subunit Rieske, mitochondrial	29.6	181.7	20.4
61	IPI00216308	VDAC1 Voltage-dependent anion-selective channel protein 1	30.8	176.0	11.0
62	IPI00003968	NDUFA9 NADH dehydrogenase [ubiquinone] 1 alpha subcomplex subunit 9, mitochondrial	42.5	170.4	14.6
63	IPI01015888	ACADM cDNA, FLJ78845, highly similar to Medium-chain specific acyl-CoA dehydrogenase, mitochondrial	42.4	170.1	14.3
64	IPI00037070	HSPA8 Uncharacterized protein	53.5	162.8	7.5
65	IPI00291006	MDH2 Malate dehydrogenase, mitochondrial	35.5	160.4	9.8
66	IPI00029133	ATP5F1 ATP synthase subunit b, mitochondrial	28.9	156.6	18.0
67	IPI00025086	COX5A Cytochrome c oxidase subunit 5A, mitochondrial	16.8	152.5	24.7
68	IPI00915869	MDH1 malate dehydrogenase, cytoplasmic isoform 3	27.0	143.4	9.0
69	IPI00081836	HIST1H2AH;HIST1H2AG;HIST1H2AK;HIST1H2AL;HIST1H2AM;HIST1H2AJ;HIST1H2AI Histone H2A type 1-H	13.9	141.9	21.9
70	IPI00029264	CYC1 Cytochrome c1, heme protein, mitochondrial	35.4	141.8	8.6
71	IPI00453473	HIST2H4B;HIST1H4C;HIST1H4J;HIST1H4D;HIST1H4A;HIST2H4A;HIST1H4I;HIST1H4K;HIST1H4E;HIST1H4L;HIST1H4F;HIST1H4H;HIST4H4;HIST1H4B Histone H4	11.4	138.4	29.1
72	IPI00216773	ALB Uncharacterized protein	45.1	132.6	7.3
73	IPI00217182	DSP Isoform DPII of Desmoplakin	260.0	128.7	2.0
74	IPI00007611	ATP5O ATP synthase subunit O, mitochondrial	23.3	127.7	10.8
75	IPI00032164	CSRP3 Cysteine and glycine-rich protein 3	21.0	124.4	9.3
76	IPI01010935	SRL cDNA FLJ51402, highly similar to Sarcalumenin	49.8	122.9	6.7
77	IPI00215777	SLC25A3 Isoform B of Phosphate carrier protein, mitochondrial	39.9	121.1	5.8
78	IPI00955815	PDHA1 pyruvate dehydrogenase E1 component subunit alpha, somatic form, mitochondrial isoform 4 precursor	40.2	119.9	5.6
79	IPI00465439	ALDOA Fructose-bisphosphate aldolase A	39.4	119.8	7.7
80	IPI00894559	NDUFA10 Uncharacterized protein	22.4	119.1	10.9
81	IPI00974544	YWHAE Isoform SV of 14-3-3 protein epsilon	26.5	117.0	12.4
82	IPI00021785	COX5B Cytochrome c oxidase subunit 5B, mitochondrial	13.7	115.9	17.8
83	IPI00719814	ATP5J2 ATP synthase subunit f, mitochondrial isoform 2d	5.7	113.1	26.5
84	IPI00895865	ETFA electron transfer flavoprotein subunit alpha, mitochondrial isoform b	30.0	112.8	14.1
85	IPI01010050	VDAC2 cDNA, FLJ78818, highly similar to Voltage-dependent anion-selective channel protein 2	27.5	111.8	9.8
86	IPI01011090	LDHB Uncharacterized protein	10.8	111.4	14.7
87	IPI00759715	FH Isoform Cytoplasmic of Fumarate hydratase, mitochondrial	50.2	110.2	7.1
88	IPI00981450	YWHAZ cDNA FLJ50142, highly similar to 14-3-3 protein zeta/delta	14.0	108.6	15.2
89	IPI00384122	DLST;DLSTP1 cDNA FLJ55034, highly similar to Dihydrolipoyllysine-residue succinyltransferase component of 2- oxoglutarate dehydrogenase complex, mitochondrial	39.5	106.8	5.4
90	IPI00004358	PYGB Glycogen phosphorylase, brain form	96.6	106.3	3.4
91	IPI00872370	NEBL Nebulette variant 4	116.6	103.6	3.5
92	IPI00946670	NDUFA5 Protein	13.1	103.2	23.2
93	IPI00556204	EEF1A2 Eukaryotic translation elongation factor 1 alpha 2 variant (Fragment)	36.9	101.4	8.2
94	IPI00217467	HIST1H1E Histone H1.4	21.9	100.9	10.5
95	IPI00815755	HIST1H2BG;HIST1H2BI;HIST1H2BE;HIST1H2BC;HIST1H2BF HIST1H2BC protein	13.8	97.6	19.0
96	IPI01011998	NDUFA8 cDNA FLJ52138, highly similar to NADH dehydrogenase (ubiquinone) 1 alpha subcomplex subunit 8	15.0	95.7	14.1
97	IPI00893108	HADHB Uncharacterized protein	37.9	95.2	9.4
98	IPI00030363	ACAT1 Acetyl-CoA acetyltransferase, mitochondrial	45.2	94.6	6.1
99	IPI00909711	VCL cDNA FLJ53006, highly similar to Vinculin	36.2	94.4	15.0
100	IPI00843765	SPTAN1 Isoform 3 of Spectrin alpha chain, brain	282.1	92.8	1.6
101	IPI00978451	CRYAB Uncharacterized protein	12.0	91.3	18.9
102	IPI00645016	S100A1 Protein S100-A1	10.5	91.0	16.0
103	IPI00980468	MAOA cDNA FLJ61220, highly similar to Amine oxidase (flavin-containing) A	44.8	91.0	2.8
104	IPI00014230	C1QBP Complement component 1 Q subcomponent-binding protein, mitochondrial	31.3	89.5	5.0
105	IPI00974274	ANXA6 Uncharacterized protein	51.7	89.2	3.5
106	IPI00925853	NDUFS1 cDNA FLJ60586, highly similar to NADH-ubiquinone oxidoreductase 75 kDa subunit, mitochondrial	66.9	85.2	6.2
107	IPI00977382	HSPA9 Protein	18.3	80.7	7.4
